# A deep learning model for identifying diabetic retinopathy using optical coherence tomography angiography

**DOI:** 10.1038/s41598-021-02479-6

**Published:** 2021-11-26

**Authors:** Gahyung Ryu, Kyungmin Lee, Donggeun Park, Sang Hyun Park, Min Sagong

**Affiliations:** 1 Department of Ophthalmology, Yeungnam University College of Medicine, #170 Hyunchungro, Nam-gu, Daegu, 42415 South Korea; 2 Yeungnam Eye Center, Yeungnam University Hospital, Daegu, South Korea; 3 Department of Robotic Engineering, DGIST, #333, Techno jungang-daero, Dalseong-gun, Daegu, South Korea

**Keywords:** Computational science, Diabetes complications, Retinal diseases, Diagnosis, Disease prevention, Health care economics, Medical imaging, Public health

## Abstract

As the prevalence of diabetes increases, millions of people need to be screened for diabetic retinopathy (DR). Remarkable advances in technology have made it possible to use artificial intelligence to screen DR from retinal images with high accuracy and reliability, resulting in reducing human labor by processing large amounts of data in a shorter time. We developed a fully automated classification algorithm to diagnose DR and identify referable status using optical coherence tomography angiography (OCTA) images with convolutional neural network (CNN) model and verified its feasibility by comparing its performance with that of conventional machine learning model. Ground truths for classifications were made based on ultra-widefield fluorescein angiography to increase the accuracy of data annotation. The proposed CNN classifier achieved an accuracy of 91–98%, a sensitivity of 86–97%, a specificity of 94–99%, and an area under the curve of 0.919–0.976. In the external validation, overall similar performances were also achieved. The results were similar regardless of the size and depth of the OCTA images, indicating that DR could be satisfactorily classified even with images comprising narrow area of the macular region and a single image slab of retina. The CNN-based classification using OCTA is expected to create a novel diagnostic workflow for DR detection and referral.

## Introduction

Diabetic retinopathy (DR) is a leading cause of blindness worldwide, and approximately 80% of patients with diabetes develop DR within 20 years of diagnosis^1–3^. Development of sight-threatening complications of DR can be delayed or prevented completely by appropriate treatment involving laser therapy or intravitreal injection of anti-vascular endothelial growth factor or steroids^4–7^. The success of these therapeutic procedures depends on timely recognition especially when the disease progresses to a stage where intervention is required. However, despite the significance of this problem and the marked increase in the prevalence of diabetes, the requirement for experts and highly trained ophthalmologists results in an expensive and time-consuming procedure^8^. In addition, the classification of DR severity and early disease detection is often subjective; thus, to a certain extent, classification often depends on expert clinical interpretation^9,10^.

To address this, several automated methods using machine learning or deep learning techniques were proposed. Most studies have applied these learning-based methods to fundus photographs to achieve high performance for various DR classification tasks, but models trained using fundus images can be limited due to vascular features deep in the retina not being represented in two-dimensional images^11–15^. Subsequently, automated methods based on optical coherence tomography (OCT) and OCT angiography (OCTA) for DR classification were proposed and also showed its feasibility^16–24^. However, DR classification method that applies deep learning using only OCTA data, not the combined data of OCT and OCTA, has not been published yet. Moreover, classification models presented to date have been trained and tested based on incomplete ground truths created on the basis of traditional fundus images that rely only on the limited angle of view without taking account retinal lesions that may be present outside the imaged area, making it difficult to evaluate its performance. As the vascular alterations caused by diabetes are widely distributed, more than 50% of DR lesions are known to be located outside seven-standard Early Treatment Diabetic Retinopathy Study (ETDRS) fields^25,26^. Previous studies reported that the presence of peripheral retinal lesions may suggest increased DR severity in 9 to 15% of eyes^26–29^.

We present an end-to-end deep convolutional neural network (CNN)-based method to detect DR and identify referable status (severe non-proliferative DR or worse) automatically from the OCTA images with more accurate ground truths using ultra-widefield (UWF) fluorescein angiography (FA). Further, we investigated the ability of the algorithm across different image sizes and retinal slabs to identify the most appropriate mode of OCTA image acquisition for DR classification. Moreover, we confirmed the feasibility of the proposed model by quantitative comparison of model performance against a machine learning-based classifier that uses handcrafted features extracted from OCTA images.

## Results

A total of 301 eyes (51 healthy normal, 51 diagnosed with diabetes mellitus (DM) without DR, 53 with mild non-proliferative DR, 49 with moderate non-proliferative DR, 48 with severe non-proliferative DR, 49 with proliferative DR) were recruited and imaged. After excluding the images with insufficient scan quality, a total of 240 datasets, consisting of 40 datasets for each stage of DR, for each of 3 × 3 mm^2^ and 6 × 6 mm^2^ scan images were obtained. For the external validation, a total of 195 eyes (33 healthy normal, 36 diagnosed with DM without DR, 34 with mild non-proliferative DR, 31 with moderate non-proliferative DR, 31 with severe non-proliferative DR, 30 with proliferative DR) were further recruited and imaged. After excluding the images with insufficient scan quality, a total of 120 datasets, consisting of 20 datasets for each stage of DR, for each of 3 × 3 mm^2^ and 6 × 6 mm^2^ scan images were obtained.

**Figure 1:**
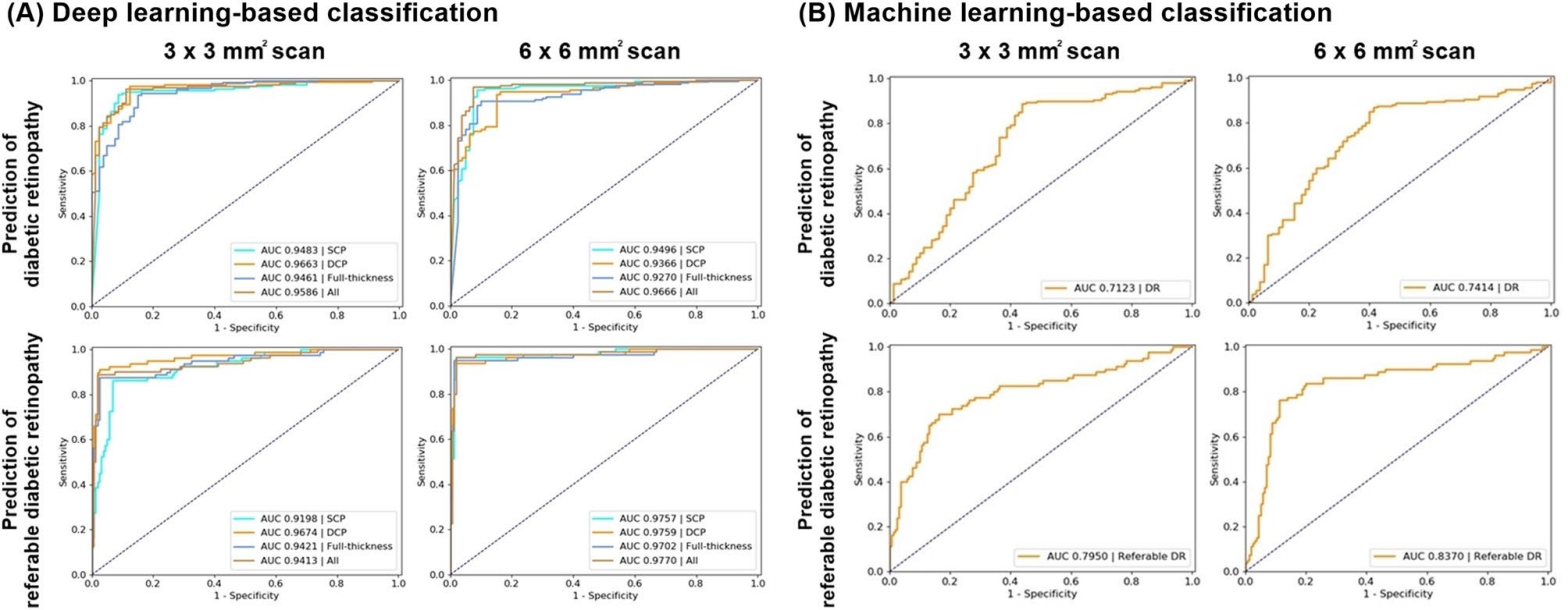
Receiver operating characteristic (ROC) curves illustrating classification performances for the prediction of onset and referable status of diabetic retinopathy (DR): (A) ROC curve using a deep learning-based classifier, and (B) ROC curve using a machine learning-based classifier. The dotted line represents the tradeoff resulting from random chance, and the solid curved line represents the tradeoff of the model. This figure was created using Photoscape (http://photoscape.co.kr/ps/main/index.php).

For the classification task of detecting the onset of DR, the area under the curves (AUC) of our CNN-based classifier using 3 × 3 mm^2^ OCTA images were measured to be 0.950 (with 95% sensitivity and 89% specificity), 0.966 (with 98% sensitivity and 88% specificity), 0.946 (with 93% sensitivity and 85% specificity), and 0.960 (with 96% sensitivity and 89% specificity) for only superficial capillary plexus (SCP), only deep capillary plexus (DCP), only full-thickness retina, and the combined data, respectively. The AUCs of the CNN-based classifier using 6 × 6 mm^2^ OCTA images were 0.949 (with 96% sensitivity and 91% specificity), 0.937 (with 95% sensitivity and 84% specificity), 0.928 (with 91% sensitivity and 90% specificity), and 0.967 (with 97% sensitivity and 93% specificity) for only SCP, only DCP, only full-thickness retina, and the combined data, respectively. Conversely, the AUCs of the machine learning-based classifier using local features extracted from OCTA images was 0.713 (with 89% sensitivity and 54% specificity) for the 3 × 3 mm^2^ images and 0.742 (with 87% sensitivity and 59% specificity) for the 6 × 6 mm^2^ OCTA images (Table 1, Fig. 1). The confusion matrix between the ground truth labels and the predictions of the proposed method are illustrated in Supplementary Fig. S1. For the external dataset, the performance of the CNN-based classifier remained at a similar level: − 0.02 to − 0.04 AUC, − 3% to − 8% sensitivity, and − 2.5% to + 8% specificity for 3 × 3 mm^2^ OCTA images, and 0 to + 0.02 AUC, 0% to − 2% sensitivity, and + 1% to + 9% specificity for 6 × 6 mm^2^ OCTA images (Table 2).

**Table 1:** Performance of classification task for detecting diabetic retinopathy using the proposed deep learning- and machine learning-based classifiers.

	Accuracy, % (SD)		Sensitivity, % (SD)		Specificity, % (SD)		AUC (SD)	
	3 × 3 mm^2^	6 × 6 mm^2^	3 × 3 mm^2^	6 × 6 mm^2^	3 × 3 mm^2^	6 × 6 mm^2^	3 × 3 mm^2^	6 × 6 mm^2^
**CNN-based classifier**								
Superficial capillary plexus layer	92.9 (4.8)	94.2 (3.6)	95.0 (2.8)	95.6 (1.8)	88.8 (11.2)	91.3 (9.0)	0.950 (0.038)	0.949 (0.031)
Deep capillary plexus layer	94.2 (1.9)	91.3 (5.3)	97.5 (4.8)	95.0 (4.5)	87.5 (13.4)	83.8 (10.3)	0.966 (0.047)	0.937 (0.047)
Full-thickness retina layer	90.4 (2.5)	90.4 (2.5)	93.1 (7.4)	90.6 (3.7)	85.0 (20.3)	90.0 (8.3)	0.946 (0.021)	0.928 (0.017)
All	93.8 (3.2)	95.4 (3.4)	96.3 (4.1)	96.9 (2.1)	88.8 (7.4)	92.5 (9.0)	0.960 (0.009)	0.967 (0.029)
**Machine learning-based classifier**								
All	77.5 (2.5)	77.5 (2.5)	89.4 (6.0)	86.9 (5.7)	53.8 (6.5)	58.8 (8.9)	0.713 (0.023)	0.742 (0.022)

*AUC* area under the curve, *CNN* convolutional neural network, *SD* standard deviation.

**Table 2:** Performance of classification task on external dataset for detecting diabetic retinopathy using the proposed deep learning- and machine learning-based classifiers.

	Accuracy, %		Sensitivity, %		Specificity, %		AUC	
	3 × 3 mm^2^	6 × 6 mm^2^	3 × 3 mm^2^	6 × 6 mm^2^	3 × 3 mm^2^	6 × 6 mm^2^	3 × 3 mm^2^	6 × 6 mm^2^
**CNN-based classifier**								
Superficial capillary plexus layer	90.8	93.3	92.5	93.8	87.5	92.5	0.931	0.949
Deep capillary plexus layer	89.2	94.2	91.3	95.0	85.0	92.5	0.918	0.956
Full-thickness retina layer	87.5	90.8	87.5	88.8	87.5	95.0	0.903	0.928
All	90.8	95.0	88.8	95.0	95.0	95.0	0.928	0.962
**Machine learning-based classifier**								
All	70.8	70.0	73.8	88.8	65.0	32.5	0.771	0.798

*AUC* area under the curve, *CNN* convolutional neural network, *SD* standard deviation.

For the classification task of diagnosing referable DR, the AUCs of the CNN-based classifier using 3 × 3 mm^2^ OCTA images were 0.919 (with 86% sensitivity and 93% specificity), 0.967 (with 91% sensitivity and 98% specificity), 0.942 (with 88% sensitivity and 98% specificity), and 0.940 (with 89% sensitivity and 97% specificity) for only SCP, only DCP, only full-thickness retina, and the combined data, respectively. The AUCs of the CNN-based classifier using 6 × 6 mm^2^ OCTA images were 0.975 (with 95% sensitivity and 98% specificity), 0.975 (with 94% sensitivity and 99% specificity), 0.970 (with 95% sensitivity and 99% specificity), and 0.976 (with 96% sensitivity and 98% specificity) for only SCP, only DCP, only full-thickness retina, and the combined data, respectively. Conversely, the AUCs of the machine learning-based classification using local features extracted from OCTA images were 0.795 (with 66% sensitivity and 86% specificity) for 3 × 3 mm^2^ images and 0.837 (with 76% sensitivity and 88% specificity) for 6 × 6 mm^2^ OCTA images (Table 3, Fig. 1). The confusion matrix between the ground truth labels and the predictions of the proposed method are illustrated in Supplementary Fig. S2. For the external dataset, the performances of the CNN-based classifier were comparable: − 0 to − 0.05 AUC, − 3% to + 9% sensitivity, and − 2% to + 5% specificity for 3 × 3 mm^2^ OCTA images, and − 0.04 to − 0.06 AUC, − 1% to − 7% sensitivity, and − 0% to − 2% specificity for 6 × 6 mm^2^ OCTA images (Table 4).

**Table 3:** Performance of classification task for detecting referable status of diabetic retinopathy using the proposed deep learning- and machine learning-based classifiers.

	Accuracy, % (SD)		Sensitivity, % (SD)		Specificity, % (SD)		AUC (SD)	
	3 × 3 mm^2^	6 × 6 mm^2^	3 × 3 mm^2^	6 × 6 mm^2^	3 × 3 mm^2^	6 × 6 mm^2^	3 × 3 mm^2^	6 × 6 mm^2^
**CNN-based classifier**								
Superficial capillary plexus layer	90.8 (4.9)	97.1 (2.6)	86.3 (6.5)	95.0 (10.6)	93.1 (5.4)	98.1 (4.7)	0.919 (0.054)	0.975 (0.024)
Deep capillary plexus layer	95.4 (1.9)	97.1 (4.1)	91.3 (5.4)	93.8 (9.4)	97.5 (4.8)	98.8 (6.5)	0.967 (0.005)	0.975 (0.020)
Full-thickness retina layer	94.2 (2.2)	97.5 (3.0)	87.5 (2.5)	95.0 (7.4)	97.5 (3.7)	98.8 (1.8)	0.942 (0.029)	0.970 (0.018)
All	94.2 (2.7)	97.5 (2.6)	88.8 (7.5)	96.3 (10.6)	96.9 (5.7)	98.1 (2.8)	0.940 (0.022)	0.976 (0.021)
**Machine learning-based classifier**								
All	79.6 (2.7)	84.2 (4.2)	66.3 (8.9)	76.3 (15.6)	86.3 (7.2)	88.2 (3.7)	0.795 (0.031)	0.837 (0.092)

*AUC* area under the curve, *CNN* convolutional neural network, *SD* standard deviation.

**Table 4:** Performance of classification task on external dataset for detecting referable status of diabetic retinopathy using the proposed deep learning- and machine learning-based classifiers.

	Accuracy, %		Sensitivity, %		Specificity, %		AUC	
	3 × 3 mm^2^	6 × 6 mm^2^	3 × 3 mm^2^	6 × 6 mm^2^	3 × 3 mm^2^	6 × 6 mm^2^	3 × 3 mm^2^	6 × 6 mm^2^
**CNN-based classifier**								
Superficial capillary plexus layer	94.2	94.2	85.0	87.5	98.8	97.5	0.907	0.909
Deep capillary plexus layer	95.8	95.0	95.0	87.5	96.3	98.8	0.938	0.921
Full-thickness retina layer	93.3	94.2	85.0	87.5	97.5	97.5	0.895	0.907
All	95.8	95.8	97.5	95.0	95.0	96.3	0.940	0.938
**Machine learning-based classifier**								
All	80.0	77.5	82.5	72.5	78.8	80.2	0.733	0.682

*AUC* area under the curve, *CNN* convolutional neural network, *SD* standard deviation.

**Figure 2:**
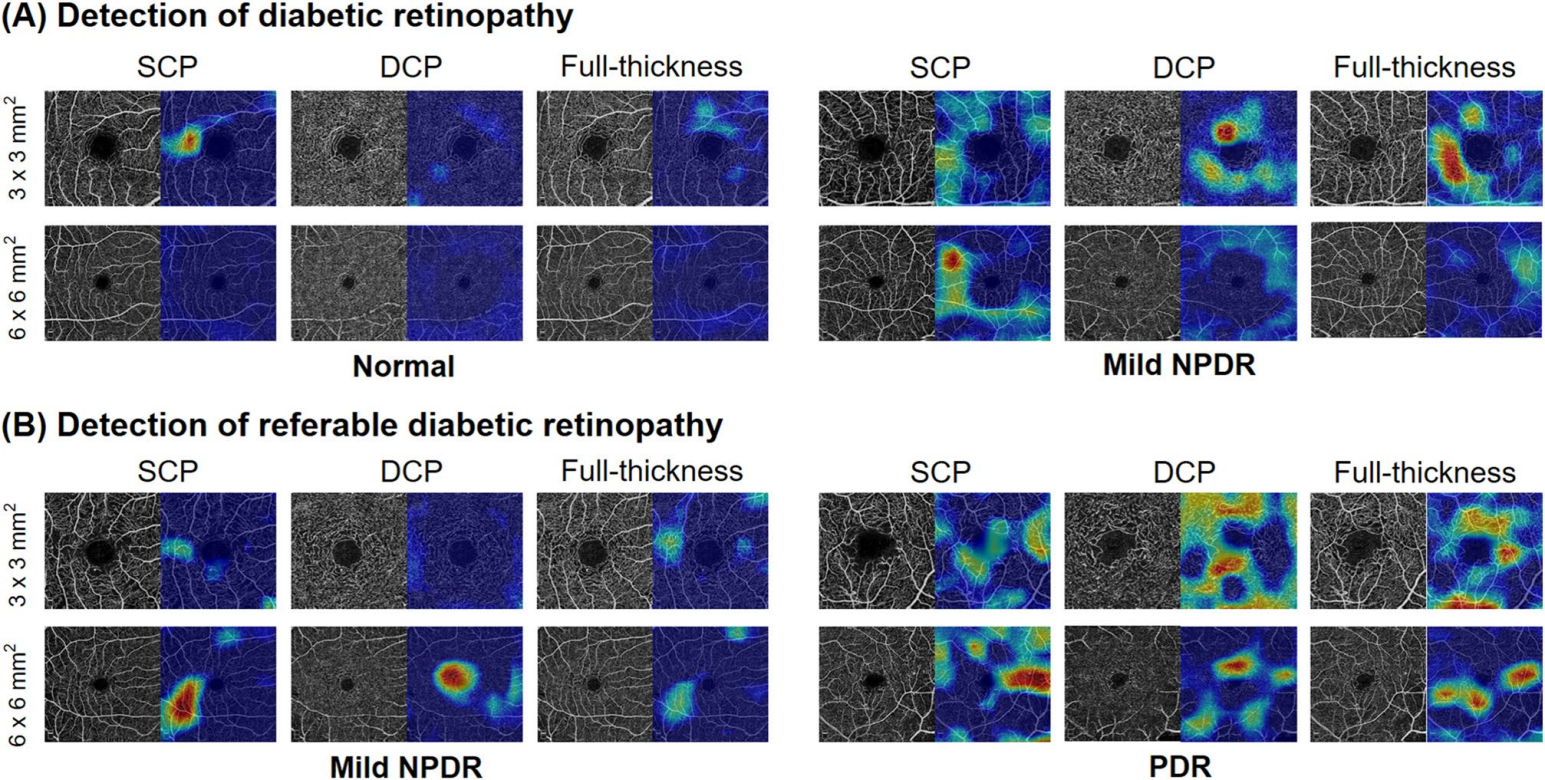
Representative Class activation map (CAM) images highlighting pathologic regions, shown in pairs of original optical coherence tomography angiography (OCTA) images (left) and corresponding CAM overlaid on the original images (right) of representative cases. CAM images were generated using the superficial capillary plexus, deep capillary plexus, and full-thickness retinal slabs from 3 × 3 mm^2^ to 6 × 6 mm^2^ macular OCTA scans. (**A**) CAM images of normal (left) and mild non-proliferative DR (right) cases for DR detection, accompanied by the corresponding original image. (**B**) CAM images of mild non-proliferative DR (left) and proliferative DR (right) for referable DR detection, accompanied by the corresponding original image. This figure was created using Photoscape (http://photoscape.co.kr/ps/main/index.php).

Figure 2 illustrates examples of OCTA images with the class activation maps (CAM) obtained using the proposed model for detecting DR and referable DR cases. In the case of no DR, the whole image was weakly activated, with the exception of the foveal avascular zone (FAZ) and the region around the large blood vessel being occasionally activated. On the other hand, in the case of referable DR, the overall area around the FAZ and vicinity of the large blood vessels were strongly activated. The activation increased in regions where the density of blood vessels significantly changed compared to regions of even spread, which is related to the non-uniformity in the blood vessel region. As the activation map visualizes areas where the network is used for decision making, rather than the specific abnormal area, the activated area was not the same for the 3 × 3 mm^2^ images and 6 × 6 mm^2^ images.

## Discussion

In this study, we designed an end-to-end deep learning-based classification system for DR and referable DR diagnoses using OCTA, based on ground truths determined clinically using UWF FA. The proposed CNN classifier produced good results with an accuracy of 90 to 95%, a sensitivity of 91 to 98%, a specificity of 85 to 93%, and an AUC of 0.93 to 0.97 for detecting the onset of DR; moreover, an accuracy of 91 to 98%, a sensitivity of 86 to 96%, a specificity of 93 to 99%, and an AUC of 0.94 to 0.98 was obtained for detecting referable DR. In the external validation, overall similar performances were also achieved. Thus, the proposed classifier showed consistently reliable performance for all OCTA slabs (whether individually or combined) including all evaluated scan sizes.

Recently, researchers have focused on automated solutions for the diagnosis and classification of retinal diseases. However, previous studies used medical records or fundus photographs with limited field of views to diagnose the stages of DR in the training set for automated models. This technique is relatively subjective which has limitations, as it cannot reflect the diabetic changes based on the entire retina. Although models based on conventional fundus photographs can be trained with large amounts of data owing to easy accessibility, there is an inherent potential for misclassification of DR. Therefore, it is uncertain whether these models will function effectively with other devices in the clinical setting. Through this study, we were able to reflect diabetic changes from the entire retina based on UWF FA for accurate diagnosis of DR in all cases, which is one of the significant advantages of this study.

In this study, a deep CNN algorithm using OCTA achieved comparable accuracy to previous CNN-based DR grading algorithms using fundus photographs with less than 250 samples^11–15^. OCTA visualizes microvascular structures in different retinal layers, enabling comprehensive quantitative analysis of pathological vascular changes relative to diabetes. As DR is primarily a disease of the retinal vasculature, OCTA can provide more instructive information than fundus photographs and perhaps a more suitable imaging modality for the automated classification of DR. This result is consistent with those of previous deep learning classification studies using OCT and OCTA, which demonstrated that the detailed information of the macular region extracted through OCT/OCTA images is sufficient to diagnose DR at a level similar to fundus photographs, although they provide limited field of view than fundus photographs^23,24^. Further studies are required to directly compare the results of deep learning algorithm using OCTA and traditional fundus photography, respectively, based on accurate ground truth using UWF FA.

Since DR can cause extensive and full-depth damages to the retinal microvasculature, we investigated the ability of the CNN algorithm across different image sizes and retinal slabs to identify which images are most appropriate for DR classification. Several cross-sectional studies have compared the diagnostic performance of DR assessment based on OCTA scan size or slab depth using quantitative microvascular metrics of OCTA, but the results are still debatable^30–39^. As those studies depended on handcrafted feature extraction for DR characterization, results may be affected by parameters of image acquisition; including oversampling density, filters and algorithms used for quantification of vascular metrics^40,41^. Moreover, though algorithms using handcrafted features may perform well on singular OCTA datasets; it can be difficult to generalize to other datasets due to overfitting on the original samples^42,43^. However, as OCTA contains more unlabeled information, a fully automated CNN algorithm can process heterogeneous images quickly regardless of the size and slab for accurate and objective DR classification, potentially alleviating the requirement for resource-intensive manual analysis and thus guiding high-risk patients for further treatment. Interestingly, we observed that the obtained results were similar regardless of the size and depth of the OCTA images. Our results showed that DR, including pathological changes of the entire retina, could be satisfactorily classified even with images comprising an area of 3 × 3 mm^2^ of the macular region and a single image slab of SCP or DCP.

The highlighted regions in the CAM images accurately correspond to the local features such as the foveal avascular zone (FAZ) area, blood vessel density, skeletal vessel density, and/or fractal dimension. In the case of no DR, the whole image was weakly activated, but only FAZ and the region around the large blood vessel were activated. Conversely, in the case of referable DR, an overall strongly activated region appeared, including the area around the FAZ and vicinity of the large blood vessels. The activation of the CAM increased in regions where the density of blood vessels significantly changed compared to regions of even spread, which is related to the non-uniformity in the blood vessel region. Based on this observation, we hypothesize that FAZ and blood vessel density played an important role in the classification of DR using CNN. As we used CAM to check how predictions are made in this study and its application to clinical practice is still a matter to be considered in the future work.

This end-to-end CNN classifier showed better efficacy than the machine learning classifier using local features extracted after the vessel and FAZ segmentation. This further indicates that traditionally known human parameters used in this study are insufficient for DR characterization and the missing critical features can be effectively extracted through end-to-end deep learning. Although it is difficult to compare the performance due to the different experimental settings, the machine-learning model in this study showed lower performance than previous studies^16–18^. The performance of the machine-learning model can be improved by increasing the dataset, but improvements may be limited even with the aforementioned features. As deep learning leverages unlabeled information to achieve the best accuracy in most cases, it can produce good results even with a small training dataset.

Though we report comparable performance in this study, a notable limitation is that the number of patients employed is still relatively small. However, the number of patients in this study is comparable to others employing OCTA^16–18^, considering that this technology is still not ubiquitous in ophthalmology practices. Additionally, the absence of FA in normal subjects may affect the reliability of the ground truth label as a whole. Although FA could not be performed in the normal subjects due to ethical issues, the presence of systemic and ophthalmic diseases was checked and excluded through detailed history taking and medical records confirmation. Lastly, although we performed external validation using additional data, it remains to be proven on a fully independent, larger, de novo set of images that also contains images with macular edema, artifacts, or low-quality for application to real-world clinical practice of this system. However, this study supports an important first step in an end-to-end deep learning models for DR classification using OCTA images. Moreover, the ground truth for classification of DR stages based on UWF FA is another strength of this study as UWF imaging involves diabetic changes from the entire retina with a wider field of view than conventional FA and fundus photography. Next, it will be necessary to elucidate the ability of the deep learning algorithm using OCTA to identify not only eyes with referrable DR but also eyes with peripheral dominant lesions or eyes with PDR.

In this work, we introduced a fully automated deep CNN DR classification method using only OCTA images. Although OCTA is rapidly adapted to the new modality in a clinical routine, the interpretation of OCTA data remains limited. If OCTA can provide comparable diagnostic value to UWF FA, invasive FA can be avoided even when diagnosis or referral decisions are difficult. In this way, the OCTA-based automated classification framework can perform more accurate DR screening than using conventional fundus photography. This system on a clinical basis is expected to drastically reduce the rate of vision loss attributed to DR, improve clinical management, and create a novel diagnostic workflow for disease detection and referral. For proper clinical application of our method, further testing and optimization of the sensitivity metrics such as genetic factors, hemoglobin A1C, duration of diabetes, and other clinical data may be required to ensure a minimum false-negative rate. Combining the data from various imaging modalities such as fundus photography or FA can reinforce the performance value, thereby further improving accuracy. Future work should include the extension of the algorithm to classify the detailed severity levels of DR with a larger number of patients.

## Methods

### Dataset

This cross-sectional study was conducted in accordance with the Declaration of Helsinki and approved by the Institutional Review Board (IRB) of Yeungnam University Medical Center (approval number: 2020-02-003). The requirement for written consent was waived by the IRB because of the retrospective nature of the study. Data were collected between January 2018 and January 2019. Data between February 2019 and January 2020 were additionally collected to validate our method on external dataset.

Subjects who had visited the hospital for visual floater and ocular discomfort, and who had undergone detailed examination including OCTA (Optovue RTVue XR AVANTI, Optovue Inc., Fremont, CA, USA), but had no systemic disease or ocular disease were retrospectively included. Subjects who had previously been diagnosed with diabetes mellitus (DM) and undergone comprehensive ophthalmic examinations including UWF FA (Optos California, Optos plc, Dunfermline, UK) and OCTA were also included. UWF FA was performed limitedly after explaining possible side effects if the patient desires a full-examination in spite of absence of DR. A small number of diabetic patients without DR wanted FA examination, accounting for less than 10% of those diabetic patients without DR who visited our clinic during the year. Only diabetic patients underwent fluorescein angiography, but not the healthy control participants. As the normal control group, patients without systemic disease who had undergone several ophthalmic examinations including mydriatic examination and OCTA for health-screening purposes, but had no definite ocular diseases were included. In the case of diabetes without retinopathy, only cases where FA was performed were included. This is retrospective study, hence, no patients received FA to participate this study. Indication of FA is not related with the protocol of this study. Exclusion criteria included the presence of glaucoma or retinal disorders affecting retinal capillary changes other than DR. Eyes with macular edema were excluded because it can obscure retinal microvasculature on OCTA. Images with low signal strength (≤ 6), excessive motion artifacts, and projection artifacts caused by media opacities were also excluded. OCTA images were obtained as volume scans of 3 × 3 mm^2^ and 6 × 6 mm^2^ sizes centered on the macula, and images of the SCP, DCP, and full-thickness retina slab were used for analysis. The ground truth for determining the accuracy of each diagnosis and grading DR was determined by two masked expert retinal specialists (G.H. and D.P.) reviewing all phases of central/axial UWF FA images, which were recorded up to 15 min after dye injection. Grading was performed based on the International Clinical DR Severity Scale^44^, which was adapted by means of extending the grading quadrants to the periphery of the entire image while maintaining the original grading nomenclature for simplicity^29,45^. When there was a disagreement between the graders, the supervising grader (M.S.) confirmed the final decision.

### Convolutional neural network-based classifier using raw images from OCTA

**Figure 3:**
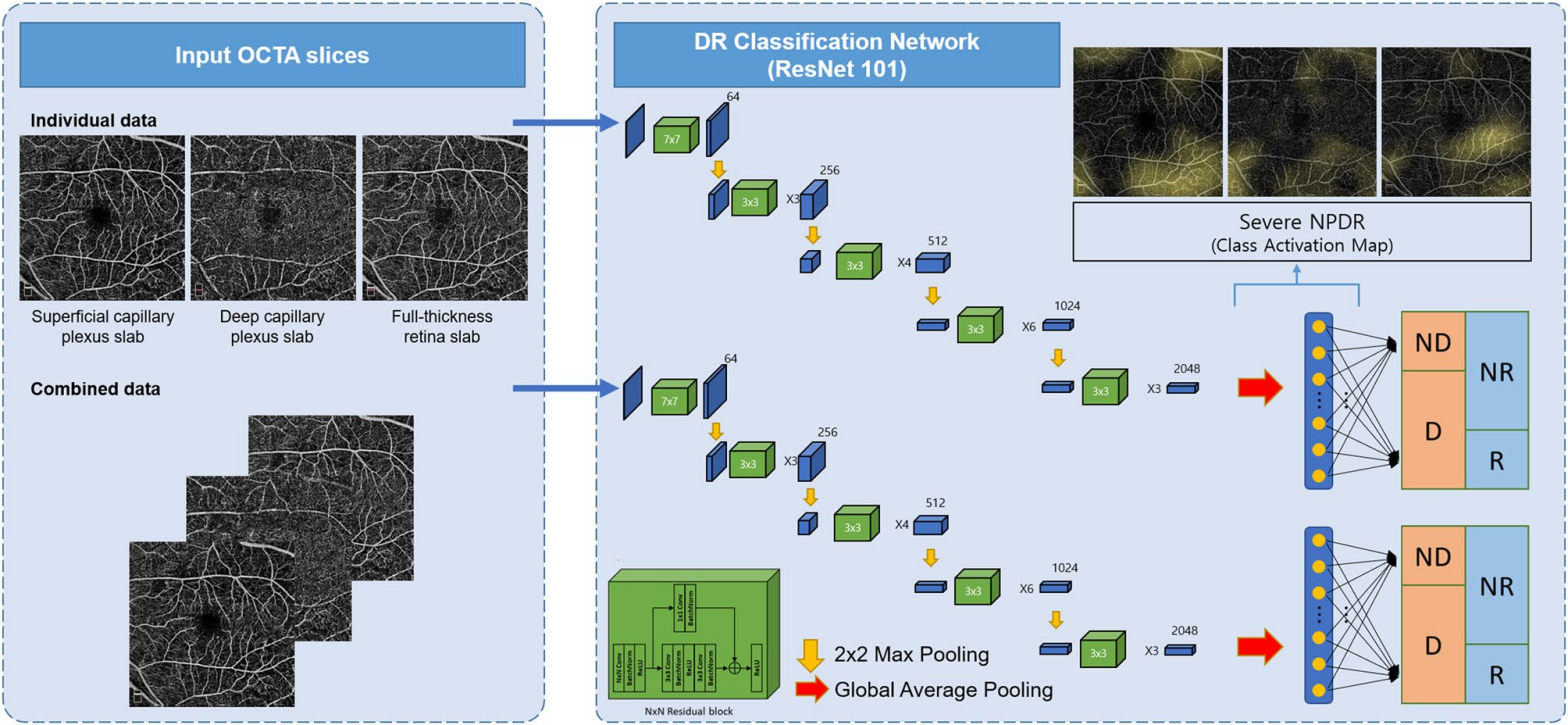
Convolutional neural network-based classifier for DR. This end-to-end classification task was performed to detect DR (D; mild non-proliferative DR or worse) and identify referable status (R; severe mild non-proliferative DR or worse) from OCTA images using a ResNet model. The OCTA images are passed through 16 residual blocks, while increasing the number of filters successively. After the residual blocks, each feature map is averaged in the global average pooling layer, and the probability for each stage is then obtained through a fully connected layer with softmax function. CAMs are derived from the global average pooling layer in the last part of the network to visualize the regions that significantly affect the tasks. This figure was created using Office Power Point software 2019 (https://www.microsoft.com/ko-kr/microsoft-365/powerpoint).

The overall structure of the proposed method for detecting early signs of DR and referable DR is shown in Fig. 3. SCP, DCP, and full-retina OCTA images were concatenated and used as input of the CNN. Using the ResNet101 model^46^, images were passed through residual blocks with 101 layers, which repeatedly performed a summation of the input and output feature maps from the convolution layers each with batch normalization, rectified linear unit (ReLU) activation functions, and max pooling. After the residual blocks, each feature map was averaged in the global average pooling (GAP) layer with the probability of each stage obtained through a fully connected layer with a softmax function. CAM were derived from the GAP layer by summating the feature maps with the weights from the last layer in order to visualize regions that show high correlation with the task of interest. It is worth nothing that parameters of the network were initially transferred from the pre-trained parameters of the ImageNet dataset, excluding the first and last layer parameters. Subsequently, all parameters were retrained using our OCTA dataset, which was optimized based on the cross-entropy loss with an Adam optimizer and a learning rate of 0.0001^47^.

### Machine learning-based classifier using local features extracted from OCTA images

**Figure 4:**
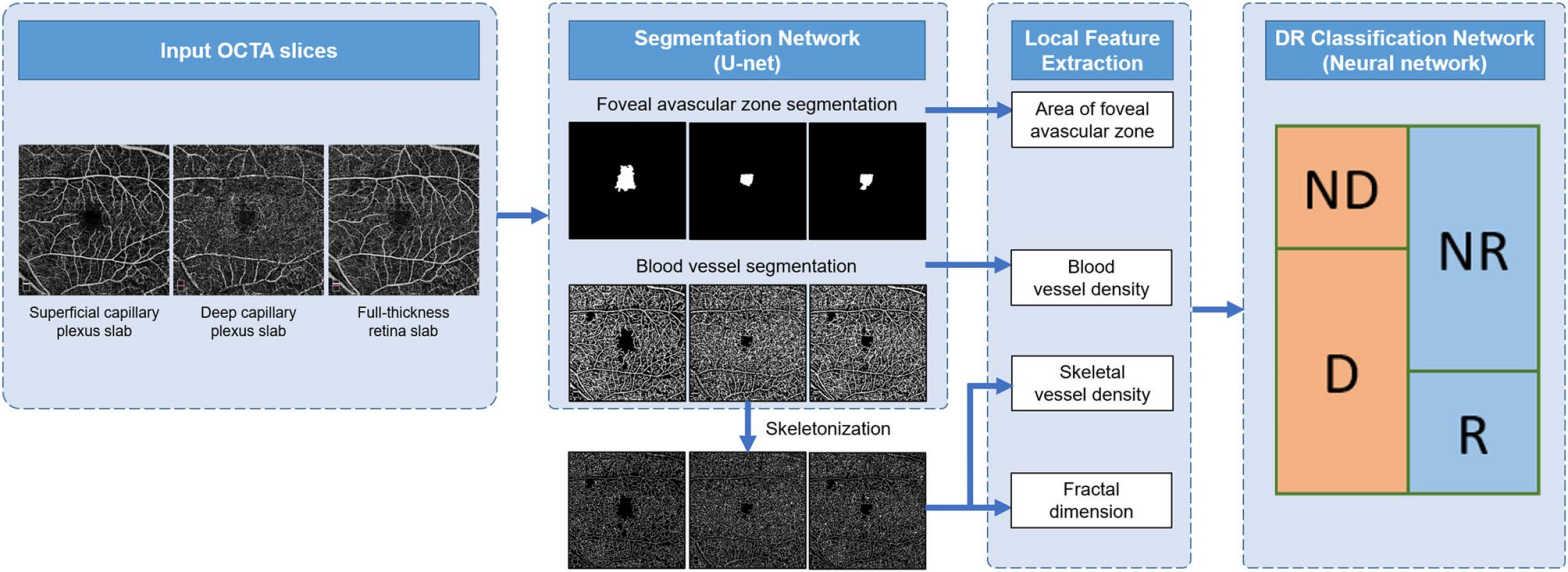
Machine learning-based classification networks for DR. Blood vessels and the FAZ were segmented from OCTA images using a U-net model. The segmented FAZ and vessels were processed to extract four significant retinal features, i.e., FAZ area, blood vessel density, skeletal vessel density, and fractal dimension. These features are supplied to a neural network classifier to detect the onset (D) and referable status (R) of DR. This figure was created using Office Power Point software 2019 (https://www.microsoft.com/ko-kr/microsoft-365/powerpoint).

The machine learning-based classifier consisted of three stages: segmentation, feature-extraction, and classification stages (Fig. 4).

In the segmentation step, U-Net^48^ was used to segment the blood vessels and FAZ from the OCTA images. The combined data from each layer of the OCTA image was used as input, given that prior machine learning based studies demonstrated that the best DR classification results were obtained when local features from the combined data was used i.e. both SCP and DCP^16–18^. In the U-Net model, a contracting path extracts high-level features from the input images by repeatedly using convolution layers, batch normalization, ReLU activation function, and max pooling; while the expanding path generates a segmentation map of the same size as the input image by repeatedly using upsampling, convolution layers, batch normalization, and ReLU activation functions on the extracted high-level features. In the expanding path, intermediate feature maps of the contracting path were concatenated with the feature maps of the previous expanding path and used as input in the next expanding path. The parameters in the network were optimized using the Adam optimizer^47^ with a dice similarity coefficient loss and learning rate of 0.0001. In the feature-extraction stage, four local features (blood vessel density, skeletal vessel density, fractal dimension, and size of the FAZ) were extracted from the segmented OCTA images. Finally, in the classification step, data from these four extracted features were fed into a neural network classifier to classify the OCTA images into DR and normal cases as well as referable DR and non-referable states.

### Experimental setting and statistical analysis

To obtain the final predictions for all the data samples, we divided the data into four distinct subsets with an even class distribution and performed four-fold cross validation. Specifically, a classifier was trained using three subsets and then tested on the remaining subset. The above operation was repeated four times with different combinations of subsets so that the DR stages of the entire dataset was obtained. The results were then compared to the ground truth determined by retinal specialists using the UWF FA images. Accuracy, sensitivity, specificity, and AUC of the system was calculated to evaluate overall performance. These metrics were calculated based on the average value obtained by four test runs. To validate our CNN method on external dataset, we trained our model using all training data used for fourfold cross validation and tested on the external dataset. The machine learning model was also trained with the same setup to compare with the CNN model.

## Supplementary Information

Supplementary Figures.

## Data Availability

The datasets generated during and/or analysed during the current study are available from the corresponding author on reasonable request.
