# Author Correction: A deep learning model for identifying diabetic retinopathy using optical coherence tomography angiography

**DOI:** 10.1038/s41598-022-25510-w

**Published:** 2022-12-05

**Authors:** Gahyung Ryu, Kyungmin Lee, Donggeun Park, Sang Hyun Park, Min Sagong

**Affiliations:** 1 Department of Ophthalmology, Yeungnam University College of Medicine, #170 Hyunchungro, Nam-Gu, Daegu, 42415 South Korea; 2 Yeungnam Eye Center, Yeungnam University Hospital, Daegu, South Korea; 3 Department of Robotic Engineering, DGIST, #333, Techno jungang-daero, Dalseong-gun, Daegu, South Korea

Correction to: *Scientific Reports* https://doi.org/10.1038/s41598-021-02479-6, published online 26 November 2021

The original version of this Article contained errors.

The description of the studied cohort was unclear in the original version of this Article, in that it did not specify that only diabetic patients underwent fluorescein angiography, but not the healthy control participants.

As a result, in Methods, subheading ‘Dataset’

“Subjects who had visited the hospital for visual floater and ocular discomfort, and who had undergone detailed examination including OCTA (Optovue RTVue XR AVANTI, Optovue Inc., Fremont, CA, USA), but had no systemic disease or ocular disease were retrospectively included. Subjects who had previously been diagnosed with diabetes mellitus (DM) and undergone comprehensive ophthalmic examinations including UWF FA (Optos California, Optos plc, Dunfermline, UK) and OCTA were also included. UWF FA was performed limitedly after explaining possible side effects if the patient desires a full-examination in spite of absence of DR. A small number of diabetic patients without DR wanted FA examination, accounting for less than 10% of those diabetic patients without DR who visited our clinic during the year. This is retrospective study, hence, no patients received FA to participate this study. Indication of FA is not related with the protocol of this study. Exclusion criteria included the presence of glaucoma or retinal disorders affecting retinal capillary changes other than DR. Eyes with macular edema were excluded because it can obscure retinal microvasculature on OCTA. Images with low signal strength (≤ 6), excessive motion artifacts, and projection artifacts caused by media opacities were also excluded. OCTA images were obtained as volume scans of 3 × 3 mm^2^ and 6 × 6 mm^2^ sizes centered on the macula, and images of the SCP, DCP, and full-thickness retina slab were used for analysis. The ground truth for determining the accuracy of each diagnosis and grading DR was determined by two masked expert retinal specialists (G.H. and D.P.) reviewing all phases of central/axial UWF FA images, which were recorded up to 15 min after dye injection. Grading was performed based on the International Clinical DR Severity Scale^44^, which was adapted by means of extending the grading quadrants to the periphery of the entire image while maintaining the original grading nomenclature for simplicity^29,45^. When there was a disagreement between the graders, the supervising grader (M.S.) confirmed the final decision.”

now reads

“Subjects who had visited the hospital for visual floater and ocular discomfort, and who had undergone detailed examination including OCTA (Optovue RTVue XR AVANTI, Optovue Inc., Fremont, CA, USA), but had no systemic disease or ocular disease were retrospectively included. Subjects who had previously been diagnosed with diabetes mellitus (DM) and undergone comprehensive ophthalmic examinations including UWF FA (Optos California, Optos plc, Dunfermline, UK) and OCTA were also included. UWF FA was performed limitedly after explaining possible side effects if the patient desires a full-examination in spite of absence of DR. A small number of diabetic patients without DR wanted FA examination, accounting for less than 10% of those diabetic patients without DR who visited our clinic during the year. Only diabetic patients underwent fluorescein angiography, but not the healthy control participants. As the normal control group, patients without systemic disease who had undergone several ophthalmic examinations including mydriatic examination and OCTA for health-screening purposes, but had no definite ocular diseases were included. In the case of diabetes without retinopathy, only cases where FA was performed were included. This is retrospective study, hence, no patients received FA to participate this study. Indication of FA is not related with the protocol of this study. Exclusion criteria included the presence of glaucoma or retinal disorders affecting retinal capillary changes other than DR. Eyes with macular edema were excluded because it can obscure retinal microvasculature on OCTA. Images with low signal strength (≤ 6), excessive motion artifacts, and projection artifacts caused by media opacities were also excluded. OCTA images were obtained as volume scans of 3  × 3 mm^2^ and 6 × 6 mm^2^ sizes centered on the macula, and images of the SCP, DCP, and full-thickness retina slab were used for analysis. The ground truth for determining the accuracy of each diagnosis and grading DR was determined by two masked expert retinal specialists (G.H. and D.P.) reviewing all phases of central/axial UWF FA images, which were recorded up to 15 min after dye injection. Grading was performed based on the International Clinical DR Severity Scale^44^, which was adapted by means of extending the grading quadrants to the periphery of the entire image while maintaining the original grading nomenclature for simplicity^29,45^. When there was a disagreement between the graders, the supervising grader (M.S.) confirmed the final decision.”

The original Article has been corrected.

