# Supplementary material for: A deep learning model for identifying diabetic retinopathy using optical coherence tomography angiography
Source: Sci Rep. 2021 Nov 26;11:23024. doi: 10.1038/s41598-021-02479-6 (PMC8626435; doi:10.1038/s41598-021-02479-6)

**Supplemental Figure 1.** Confusion matrix for detecting the onset of diabetic retinopathy.


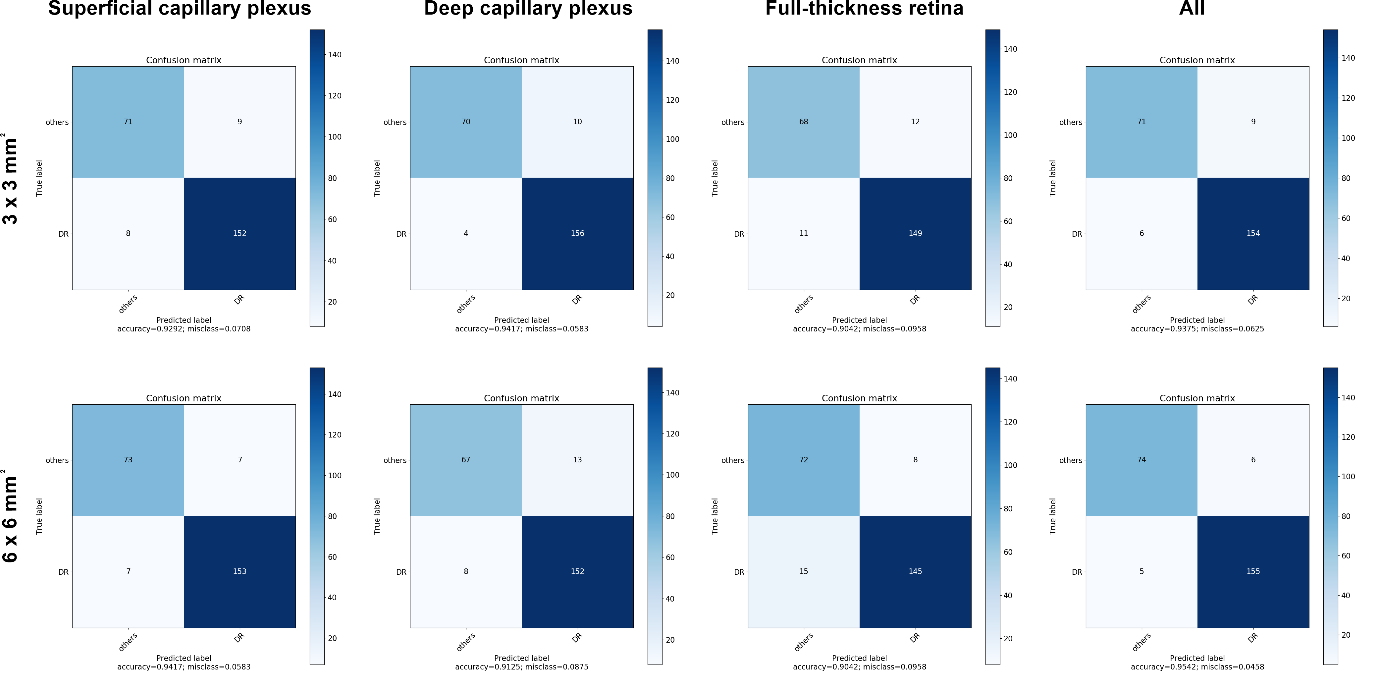


**Supplemental Figure 2.** Confusion matrix for diagnosing referable diabetic retinopathy.


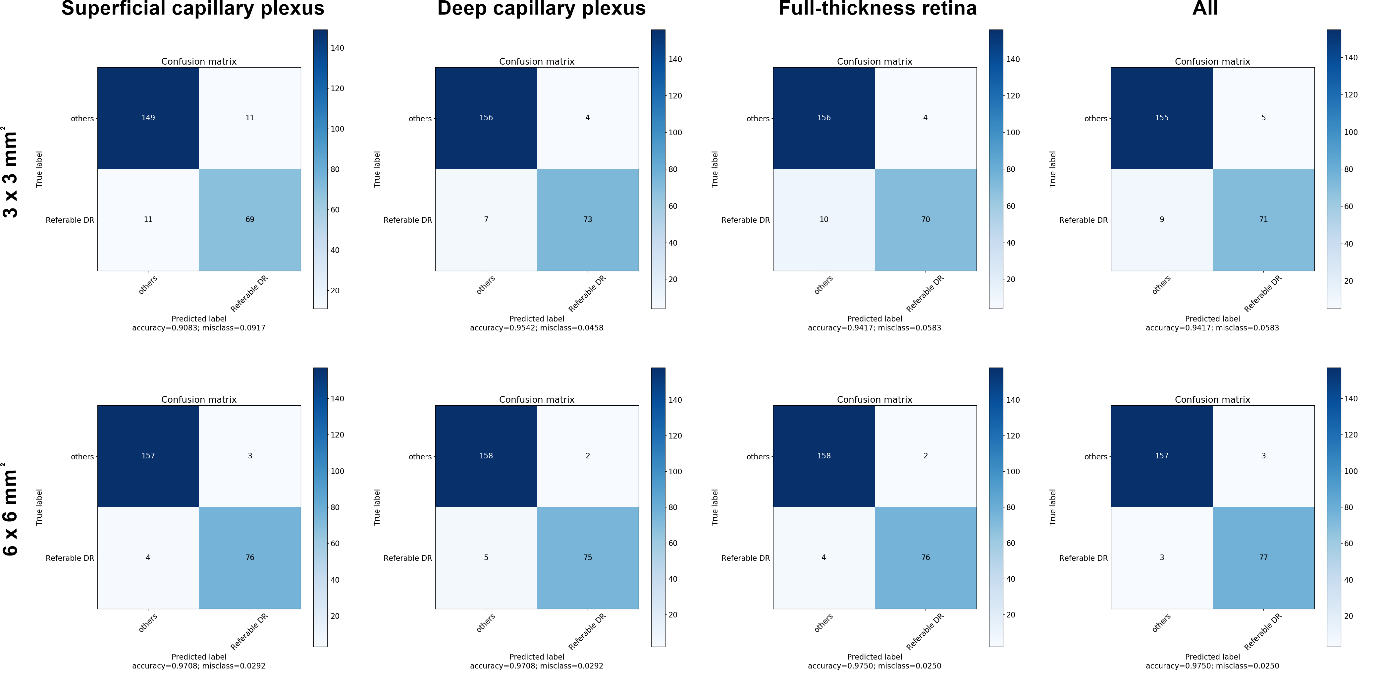

Supplement: Supplementary file 1 — Supplementary Figures. [file 41598_2021_2479_MOESM1_ESM.docx]
